# Supplementary figures and images for: Clinical and prognostic value of preoperative hydronephrosis in upper tract urothelial carcinoma: a systematic review and meta-analysis
Source: PeerJ. 2016 Jun 21;4:e2144. doi: 10.7717/peerj.2144 (PMC4924132; doi:10.7717/peerj.2144)

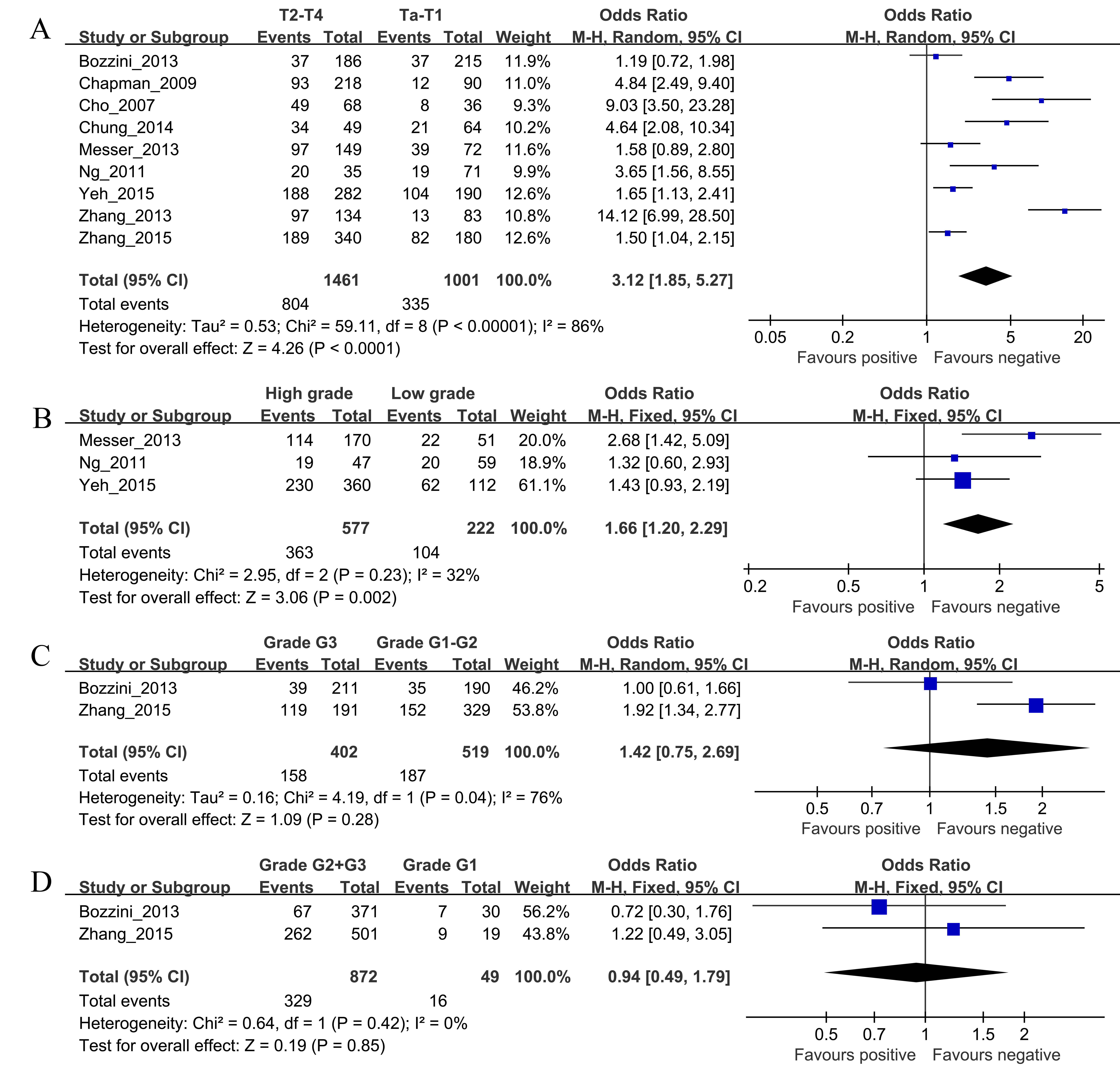

Supplement: Figure S1 — (A) The odds ratio (OR) of preoperative hydronephrosis associated with tumor stage in UTUC patients (Ta/1 vs T2-4); (B) The odds ratio (OR) of preoperative hydronephrosis associated with tumor grade in UTUC patients (High grade vs. Low grade); (C) The odds ratio (OR) of preoperative hydronephrosis associated with tumor grade in UTUC patients (G3 vs. G1+G2); (D) The odds ratio (OR) of preoperative hydronephrosis associated with tumor grade in UTUC patients (G2 + G3 vs. G1). [file peerj-04-2144-s001.png]

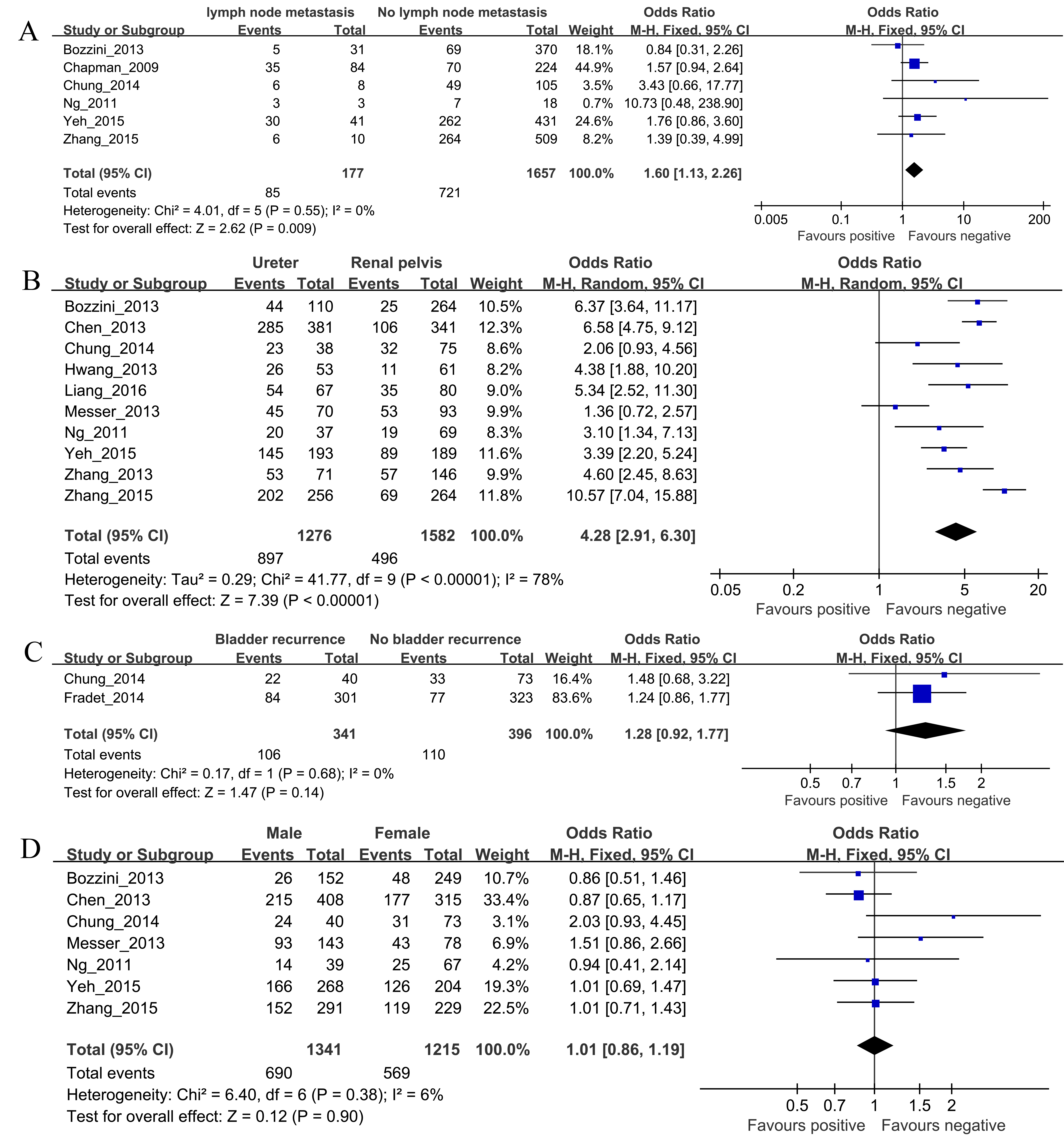

Supplement: Figure S2 — (A) The odds ratio (OR) of preoperative hydronephrosis associated with lymph node status in UTUC patients (Lymph node metastasis vs. No lymph node metastasis); (B) The odds ratio (OR) of preoperative hydronephrosis associated with tumor location in UTUC patients (Renal pelvis vs. Ureter); (C) The odds ratio (OR) of preoperative hydronephrosis associated with recurrence status in UTUC patients (Recurrence vs. No recurrence); (D) The odds ratio (OR) of preoperative hydronephrosis associated with gender in UTUC patients (Male vs. Female). [file peerj-04-2144-s002.png]

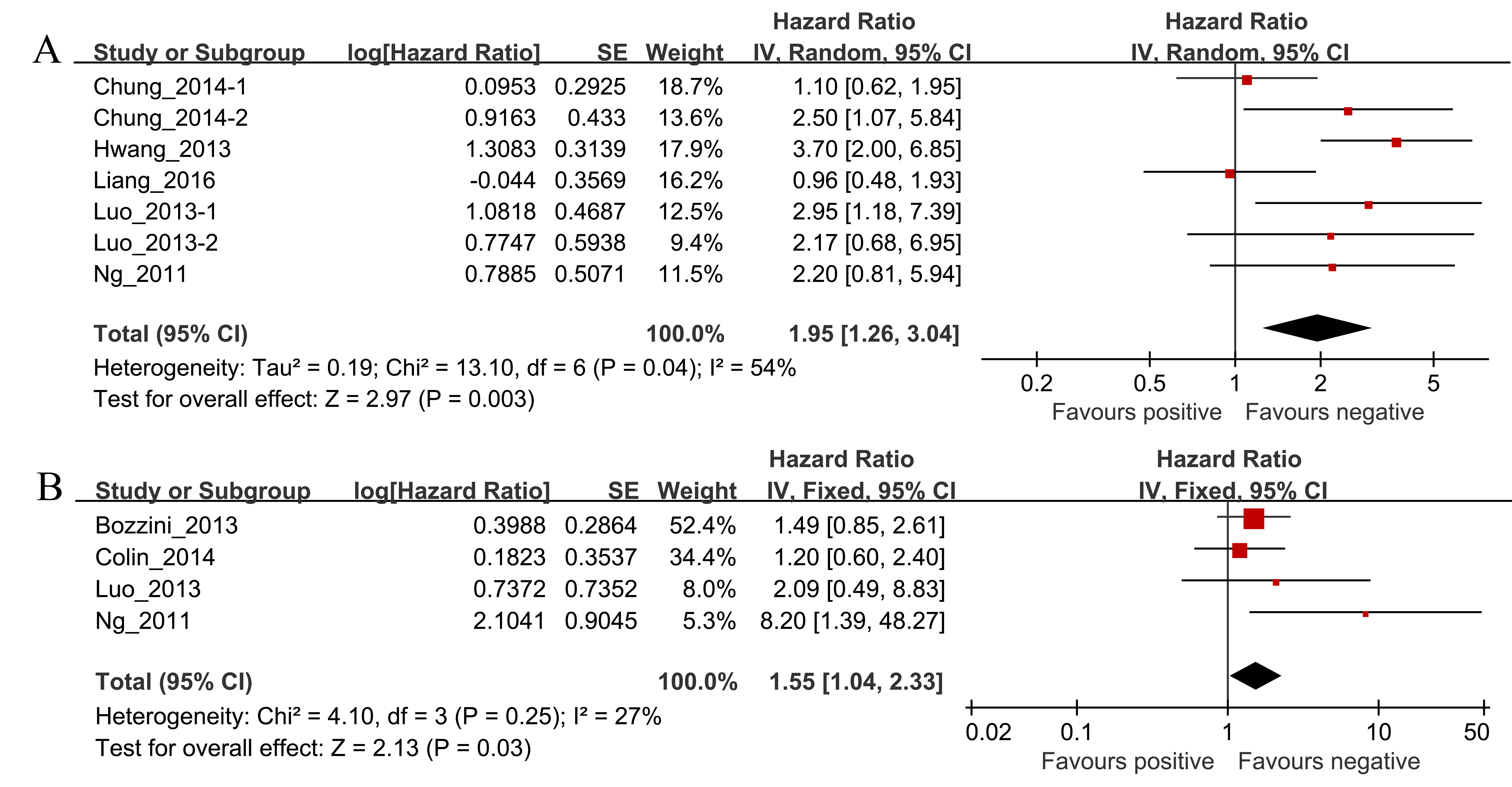

Supplement: Figure S3 — (A) The hazard ratio (HR) of associated with RFS in UTUC patients; (B) The hazard ratio (HR) of associated with MFS in UTUC patients. [file peerj-04-2144-s003.png]
