# Supplementary material for: Clinical and prognostic value of preoperative hydronephrosis in upper tract urothelial carcinoma: a systematic review and meta-analysis
Source: PeerJ. 2016 Jun 21;4:e2144. doi: 10.7717/peerj.2144 (PMC4924132; doi:10.7717/peerj.2144)
Supplement: Table S1 [file peerj-04-2144-s004.docx]

**Supplement Table 1. Estimation of the hazard ratio**

| **Study** | **Survival** | **Estimated** | **Survival analysis** | **Covariates** | **CSS/OS/RFS/MFS HR (95%CI)** |
| --- | --- | --- | --- | --- | --- |
| Cho_2007 | NA | NA | NA | NA | NA |
| Chapman_2009 | OS | No | Multivariate analysis | Margin status, pT stage, LN | O: 1.7(1.2-2.5) |
| Ng_2011 | CSS, RFS, MFS | No | Multivariate analysis | Age, gender, tumor location, ureteroscopic biopsy grade, urinary cytology | C: 12.1(1.27-114.99)  R: 2.2(0.81-5.94)　 M: 8.2(1.39-48.27) |
| Messer_2013 | NA | NA | NA | NA | NA |
| Bozzini_2013 | CSS, OS,  MFS | No | Univariate and multivariate analysis | Age, pT stage, LN, LVI, tumor grade, tumour location, margin status | C: 1.19(0.53–2.64)  O: 1.27(0.69–2.35) M: 1.49(0.85–2.62) |
| Zhang_2013 | CSS, PFS | No | Univariate and multivariate analysis | Age, gender, type of surgery, pT stage, LN, tumor grade, LVI, tumour location | C: 5.3 (3.2–8.8) |
| Hwang_2013 | RFS | No | Multivariate analysis | Diabetes mellitus, pT stage | R: 3.7(2.0-6.5) |
| Luo_2013 | RFS, MFS | No | Multivariate analysis | Bladder cancer history, non-organ-confined disease, gender, multifocality | R: 2.95(1.18-7.41) and 2.17(0.68-6.85)  M: 2.09(0.50-8.77) |
| Sakano_2013 | CSS | No | Univariate and multivariate analysis | Tumor location, multifocality, pT stage, voided urine cytology, neoadjuvant chemotherapy, hemoglobin, white blood cell, C-reactive protein | C: 1.20(0.85–1.72) |
| Chen_2013 | NA | NA | NA | NA | NA |
| Zou_2014 | CSS | No | Univariate and multivariate analysis | Type of surgery, gender, age, previous bladder cancer, laterality, tumor location, tumor size, appearance, necrosis, pT stage, tumor grade, multifocality, concomitant CIS, margin status, LVI | C: 2.38(0.35-1.87) |
| Colin_2014 | MFS | No | Univariate analysis | NA | M: 1.20(0.60–2.38) |
| Fradet_2014 | NA | NA | NA | NA | NA |
| Chung_2014 | CSS, RFS | No | Univariate and multivariate analysis | Tumor location, tumor architecture, tumor grade, pT stage, LVI | C: 2.6(1.05–6.22)  R: 1.1(0.62-2.02) and 2.5(1.07-5.64) |
| Yeh_2015 | CSS, OS | No | Univariate and multivariate analysis | Age, gender, smoking status, preoperative eGFR, type of surgery, tumor location, pT stage, LN, tumor grade, adjuvant chemotherapy, hematuria, flank pain | C: 1.14(0.71-1.84) and 1.87(1.04-3.37)  O: 1.15(0.73-1.80) and 1.97(1.14-3.43) |
| Zhang_2015 | CSS, OS | No | Multivariate analysis | Age, gender, tumor architecture, pT stage, tumor grade,tumor necrosis | C: 1.93(1.29-2.88)  O: 1.59(1.11-2.26) |
| Liang_2016 | CSS, OS, RFS | No | Univariate and multivariate analysis | Age, multifocality, pT stage, previous or synchronous NMIBC, tumor grade, LN, LVI, adjuvant therapy | C: 1.71(0.91-3.20);  O: 2.57(1.46-4.50)  R: 0.96(0.48-1.93) |
| Xing_2016 | CSS | No | Univariate analysis | NA | C: 1.73(0.87-3.44) |
| Zhang_2016 | CSS | No | Multivariate analysis | Gender, ureter involvement, tumor architecture, pT stage, LN, tumor grade, plasma fibrinogen | C: 0.96(0.51-1.80) |

**Notes.**

Abbreviations: CI = confidence interval; CSS = cancer-specific survival; HR = hazard ratio; LN = lymph node; LVI = lymphovascular invasion; MFS = metastasis-free survival; NA = not available; NMIBC = nonmuscle-invasive bladder cancer; OS = overall survival.
