# Supplementary material for: Clinical and prognostic value of preoperative hydronephrosis in upper tract urothelial carcinoma: a systematic review and meta-analysis
Source: PeerJ. 2016 Jun 21;4:e2144. doi: 10.7717/peerj.2144 (PMC4924132; doi:10.7717/peerj.2144)
Supplement: Table S2 [file peerj-04-2144-s005.docx]

**Supplement Table 2. Preoperative hydronephrosis according to pathological features.**

| Study | pT stage | | | | | pN stage | | | Tumor grade | | |
| --- | --- | --- | --- | --- | --- | --- | --- | --- | --- | --- | --- |
|  | pTa/is | pT1 | pT2 | pT3 | pT4 | pNx | pN0 | pN+ | G1 | G2 | G3 |
| Cho_2007 | 8/36(Ta/T1) | | 17/30 | 32/38(T3/ T4) | | NA | NA | NA | NA | NA | NA |
| Chapman_2009 | 12/90(Ta/Tis/T1) | | 26/61 | 38/100 | 29/57 | NA | 70/224 | 35/84 | NA | NA | NA |
| Ng_2011 | 15/52 | 4/19 | 7/14 | 10/17 | 3/4 | NA | 7/18 | 3/3 | NA | NA | NA |
| Messer_2013 | 39/72(Ta/Tis/T1) | | 97/149(T2/T3/ T4) | | | NA | NA | NA | NA | NA | NA |
| Bozzini_2013 | 21/121 | 16/94 | 12/37 | 23/122 | 2/27 | 30/116 | 39/254 | 5/31 | 7/30 | 28/160 | 39/211 |
| Zhang_2013 | 1/20 | 3/41 | 17/22 | 31/57 | 5/6 | NA | NA | NA | NA | NA | NA |
| Hwang_2013 | NA | NA | NA | NA | NA | NA | NA | NA | NA | NA | NA |
| Luo_2013 | NA | NA | NA | NA | NA | NA | NA | NA | NA | NA | NA |
| Sakano_2013 | NA | NA | NA | NA | NA | NA | NA | NA | NA | NA | NA |
| Chen_2013 | NA | NA | NA | NA | NA | NA | NA | NA | NA | NA | NA |
| Zou_2014 | NA | NA | NA | NA | NA | NA | NA | NA | NA | NA | NA |
| Colin_2014 | NA | NA | NA | NA | NA | NA | NA | NA | NA | NA | NA |
| Fradet_2014 | NA | NA | NA | NA | NA | NA | NA | NA | NA | NA | NA |
| Chung_2014 | 21/64(Ta/Tis/T1) | | 34/49(T2/T3/ T4) | | | 19/28 | 30/47 | 6/8 | NA | NA | NA |
| Yeh_2015 | 38/60 | 66/130 | 73/112 | 95/142 | 20/28 | 166/261 | 96/170 | 30/41 | NA | NA | NA |
| Zhang_2015 | 8/30 | 74/150 | 127/202 | 59/135 | 3/3 | 31/51 | 233/458 | 6/10 | 9/19 | 143/310 | 119/191 |
| Liang_2016 | NA | NA | NA | NA | NA | NA | NA | NA | NA | NA | NA |
| Xing_2016 | NA | NA | NA | NA | NA | NA | NA | NA | NA | NA | NA |
| Zhang_2016 | NA | NA | NA | NA | NA | NA | NA | NA | NA | NA | NA |

**Notes.**

Abbreviations: NA = not available.
