# Supplementary material for: Clinical and prognostic value of preoperative hydronephrosis in upper tract urothelial carcinoma: a systematic review and meta-analysis
Source: PeerJ. 2016 Jun 21;4:e2144. doi: 10.7717/peerj.2144 (PMC4924132; doi:10.7717/peerj.2144)
Supplement: Table S3 [file peerj-04-2144-s006.docx]

**Supplement Table 3. Preoperative hydronephrosis according to tumor features.**

| **Study** | **Gender** | | **Tumor location** | | **Bladder recurrence** | |
| --- | --- | --- | --- | --- | --- | --- |
|  | **Male** | **Female** | **Renal pelvis** | **Ureter** | **Positive** | **Negative** |
| Cho_2007 | NA | NA | NA | NA | NA | NA |
| Chapman_2009 | NA | NA | NA | NA | NA | NA |
| Ng_2011 | 25/67 | 14/39 | 19/69 | 37 | NA | NA |
| Messer_2013 | 43/78 | 93/143 | 53/93 | 45/70 | NA | NA |
| Bozzini_2013 | 48/249 | 26/152 | 25/264 | 44/110 | NA | NA |
| Zhang_2013 | NA | NA | 57/146 | 53/71 | NA | NA |
| Hwang_2013 | NA | NA | 11/61 | 26/53 | NA | NA |
| Luo_2013 | NA | NA | NA | NA | NA | NA |
| Sakano_2013 | NA | NA | NA | NA | NA | NA |
| Chen_2013 | 177/315 | 215/408 | 106/341 | 285/381 | NA | NA |
| Zou_2014 | NA | NA | NA | NA | NA | NA |
| Colin_2014 | NA | NA | NA | NA | NA | NA |
| Fradet_2014 | NA | NA | NA | NA | 84/301 | 77/323 |
| Chung_2014 | 177/315 | 215/408 | 31/65 | 24/48 | 22/40 | 33/73 |
| Yeh_2015 | 126/204 | 166/268 | 89/189 | 145/193 | NA | NA |
| Zhang_2015 | 119/229 | 152/291 | 69/264 | 202/256 | NA | NA |
| Liang_2016 | NA | NA | 35/80 | 54/67 | NA | NA |
| Xing_2016 | NA | NA | NA | NA | NA | NA |
| Zhang_2016 | NA | NA | NA | NA | NA | NA |

**Notes.**

Abbreviations: NA = not available.
